# Variable Temperature Spectroscopic Ellipsometry as a Tool for Insight into the Optical Order in the P3HT:PC70BM and PC70BM Layers

**DOI:** 10.3390/polym15183752

**Published:** 2023-09-13

**Authors:** Barbara Hajduk, Paweł Jarka, Henryk Bednarski, Tomasz Tański

**Affiliations:** 1 Centre of Polymer and Carbon Materials, Polish Academy of Sciences, 34 Marie Curie-Skłodowska Str., 41-819 Zabrze, Poland; hbednarski@cmpw-pan.pl; 2 Department of Engineering Materials and Biomaterials, Silesian University of Technology, Konarskiego 18a, 44-100 Gliwice, Poland; pawel.jarka@polsl.pl (P.J.); tomasz.tanski@polsl.pl (T.T.)

**Keywords:** variable temperature spectroscopic ellipsometry, organic semiconductors, polymer films, ellipsometric modeling, dielectric function

## Abstract

Two combined ellipsometric techniques—variable angle spectroscopic ellipsometry (VASE) and variable temperature spectroscopic ellipsometry (VTSE)—were used as tools to study the surface order and dielectric properties of thin films of a poly(3-hexylthiophene-2,5-diyl) (P3HT) mixture with a fullerene derivative (6,6-phenyl-C71-butyric acid methyl ester) (PC70BM). Under the influence of annealing, a layer of the ordered PC70BM phase was formed on the surface of the blend films. The dielectric function of the ordered PC70BM was determined for the first time and used in the ellipsometric modeling of the physical properties of the P3HT:PC70BM blend films, such as their dielectric function and thickness. The applied ellipsometric optical model of the polymer–fullerene blend treats the components of the blend as a mixture of optically ordered and disordered phases, using the effective medium approximation for this purpose. The results obtained using the constructed model showed that a layer of the ordered PC70BM phase was formed on the surface of the layer of the polymer and fullerene mixture. Namely, as a result of thermal annealing, the thickness of the layer of the ordered fullerene phase increased, while the thickness of the underlying material layer decreased.

## 1. Introduction

In recent years, functional organic materials and nanostructures have gained great importance, primarily in the production of electronics with advanced electrical and optical properties and flexibility [1,2,3,4]. Flexible electronic devices, like organic photovoltaic cells (OPV), organic light-emitting diodes (OLED), organic field-effect transistors (OFET) and integrated systems have a high importance for development of computer devices, applicable in such industries as medicine and automotive [5,6,7,8]. The importance of active organic materials in modern electronics grew rapidly with the development of multilayer devices based on low-molecular amorphous compounds in the 1980s [9].

The use of semiconductor active layers of the polymer–fullerene type produced in this process provides one of the highest reported efficiencies for third-generation photovoltaic cells. The use of the active layer in the form of a combination of electron acceptor and donor polymers poly(3-hexylthiophene-2,5-diyl) (P3HT) and phenyl-C61(also 71)-butyric acid methyl ester (PCBM) [10,11,12,13,14,15,16,17] allowed obtaining of high-efficiency OPVs due to efficient electron absorption and extended near-infrared absorption [18], what can be found in review article [17]. Better performance of organic cells, compared to standard ones based on P3HT and PCBM, can be achieved by replacing either the donor or acceptor with new materials—for example, replacing PCBM with new, non-fullerene materials, such as Zy-4Cl [15] or IDTBTC8-CN [16]. Similarly, high efficiency values were demonstrated for thin-film devices with active layers PTB7–PC70BM and PCDTBT–PC70BM [19,20,21,22,23]. Widely described research on the production of thin-film electronic devices (including photovoltaic systems) is based on the selection of component materials, their effective joining and homogenization, and the development of a technology for the deposition of continuous homogeneous ultra-thin layers. The microstructure of the active organic layers significantly affects the physical properties of the devices in which they are used. There is a clear relationship between the microstructure of the active layers and the performance of these devices [24,25,26]. Therefore, it is very important to control and optimize the morphology of these layers in technological processes. Materials such as P3HT, PC60BM, and PC70BM tend to organize and crystallize in layers, resulting in appearing of aggregation areas [27,28].

The aim of this article is to show how the ordered PC70BM phase forms on the surface of P3HT–PC70BM blend films. The novelty in this work is the determination of PC70BM’s dielectric function components for the first time. Similarly, as in the work of Bednarski et al. [29], where the morphology of P3HT–PC60BM layers was investigated, here, the polymer–fullerene films are treated as a mixture of low- and high-ordered P3HT and amorphous and ordered PC70BM phases.

In this case, we use two connected ellipsometric techniques—VASE and VTSE [30,31]—which enable the observation and control of the morphology of such layers. The main advantage is fact these are non-destructive and non-invasive measurements. We obtain the results only by measuring the polarization change of light beam, reflected from the surface of the tested samples. The ellipsometric techniques enable us to obtain information about many physical properties of the tested samples, such as the thickness, refractive index, extinction coefficient, dielectric constants, or even thermal transformation temperatures, like the glass transition temperature T_g_, cold crystallization temperature T_cc_, and melting point T_m_. The determination of thermal transitions using VTSE is possible in two ways: using temperature variations of physical parameters, like thickness d, refractive index n, or thermal expansion coefficient α, and using temperature changes of raw data, like ellipsometric angles Ψ and Δ or their temperature derivatives [32,33,34].

In our earlier works, VTSE was used in correlation with differential scanning calorimetry (DSC) [35,36,37] and temperature resistance measurements [38], as alternative, confirmative methods. In the work of Mei and Chung, it was shown that electrical resistance can be useful in studying the thermal properties and thermal history of carbon-fiber-reinforced nylon-6 composites [39]. They proved that thermal treatment influenced the T_g_ and T_m_ of the material and showed that this measurement method is more sensitive for thermal transitions than DSC, used as a reference technique.

In this work, we use the VASE and VTSE tools to study films made of a mixture of polymers and fullerenes in order to examine how the thickness of the P3HT–PC70BM and PC70BM layers changes under the influence of thermal treatment.

## 2. Experimental

**Figure 1:**
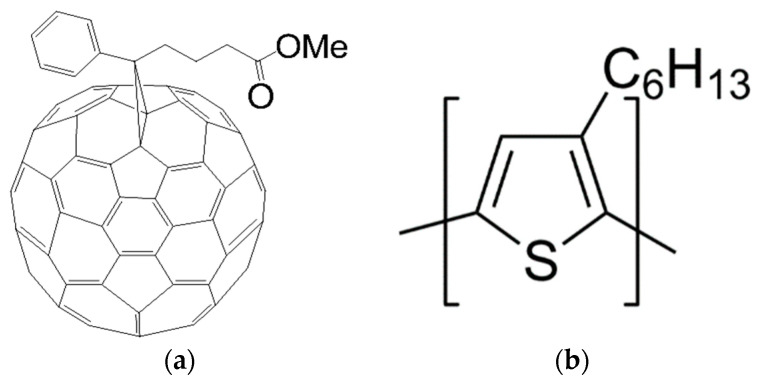
Structures of PC70BM (**a**) and P3HT (**b**).

The material used was 99 wt% purity [6,6]-phenyl-C71-butyric acid methyl ester, M114-PCBM or PC70BM, with molar mass Mw = 1031 g/mol, and 95.7 wt% purity regioregular poly(3 hexylthiophene-2,5-diyl) M102-P3HT, which were supplied by Ossila (Sheffield, UK). Their chemical structures are shown in Figure 1.

The neat materials and their blend were dissolved in chloroform. The concentrations of solutions were constant and equal to 20 mg/mL. The prepared solutions (neat P3HT, neat PC70BM, and their 1:1 blend) were stirred for 24 h at 60 °C. Thin single films of P3HT, PC70BM, and their 1:1 mixtures were deposited on silicon substrates coated with SiO_2_ of various thicknesses (90, 300, and 400 nm) by spin-coating. The spinning rate was 1000 rpm and the spinning time was 60 s. The prepared samples were kept in a laboratory dry box at room temperature.

All the films were measured using two combined ellipsometric techniques—variable angle spectroscopic ellipsometry and variable temperature spectroscopic ellipsometry. The spectra of ellipsometric angles Ψ and Δ were recorded in the wavelength range 240–2500 nm, at incidence angles 40–70° with a step of 10° and at the temperature values 25, 50, 70, 90, 110, 130, 150, 170, 190, and 210 °C under atmospheric conditions. After each measurement, an image of the surface was taken using an optical microscope, built into the ellipsometer. In all attached pictures, the diameter of the viewing circle is equal to 1 mm.

Ellipsometric measurements were performed using a SENTECH SE850E spectroscopic ellipsometer, equipped with a variable temperature vacuum chamber, operating at low pressures, and an INSTEC mK1000 temperature controller. The ellipsometer was operated with the Spectra Ray 3 software [40]. The detection limits of the thin layer thickness for the VASE and VTSE techniques are determined on the basis of the accuracy of the measurement of the ellipsometric angles Ψ and Δ, which is 0.05 and 0.2 degrees, respectively, and the properties of the tested optical system. Therefore, these limits of detection are not rigid. Nevertheless, they are usually not worse than 10^−2^ for the refractive index and a few tenths of a nanometer for the layer thickness. Importantly, they can be significantly improved by the appropriate (optimal) selection of the experimental conditions, such as the type of substrate, spectral range, angle of incidence of light, layer thickness, or sample quality.

## 3. Results and Discussion

The aim of this work was to determine the dielectric function of the low- and high-ordered phases of the PC70BM fullerene derivative. To this end, we combined two ellipsometric techniques—VASE and VTSE. The first one allows measurements of ellipsometric angles Ψ and Δ, at variable incident light angles. In this mode, it is possible to develop an accurate optical model by fitting its parameters to the experimentally determined ellipsometric angles Ψ and Δ at the lowest mean squared error (MSE). The second mode allows us to measure Ψ and Δ under thermal annealing, in heating or cooling cycles alternatively. Usually, this mode is used for thermal transition investigations, in low vacuum conditions, as in our earlier works [35,36,37,38]. In this study, due to the need to monitor the surface changes, with a built-in optical microscope, all measurements were made in atmospheric air conditions. The sample under investigation was heated to the specified temperature values, listed in the experimental section, and then an angular measurement was performed. The surface images were taken after every measurement.

**Figure 2:**
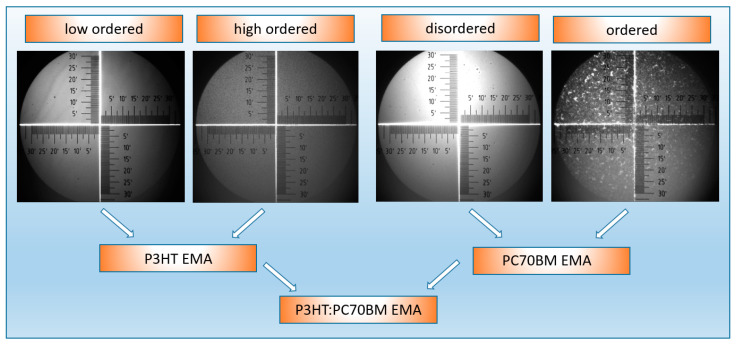
Optical model of P3HT and PC70BM, included in ellipsometric model of P3HT–PC70BM blend, with added photos of neat P3HT and PCBM films’ surfaces, before and after annealing.

It is known from the literature that the layers of the P3HT–PC70BM mixture crystallize under the influence of thermal annealing [41] and can influence the performance of organic solar cells [42]. P3HT is a liquid crystal polymer; therefore, its films are never 100% amorphous. In this case, the structure of the polymer is always more or less ordered. In turn, the PC70BM layers are more amorphous; hence, we distinguish here between the disordered and ordered phases of these materials. Since we used optical methods to identify them, we discuss optically ordered and disordered phases. PCBM nanocrystals are present in thin PCBM layers even without annealing [29,30]. At the same time, the annealing of these layers at high temperatures causes the appearance of large crystalline precipitates on their surface. Therefore, to determine the dielectric function of the ordered and disordered PC70BM phases on the basis of the effective medium approximation (EMA), layers annealed to a temperature not higher than 150 °C were used, analogically to [29]. The assumed optical models for neat materials are shown in Figure 2. They take into account the presence of an ordered and disordered phase within EMA. These models therefore describe homogeneous materials.

The dielectric function of P3HT and PC70BM as well as their 1:1 blend (P3HT:PC70BM) was described using EMA of the Bruggeman type [29,40,43,44]. In this approximation, the dielectric function of the effective medium $\varepsilon_{e}$ is determined from the following equation [29,40]:

(1) $$0\equiv f_{1}\left(\varepsilon_{i1}-\varepsilon_{e}\right){\left(\varepsilon_{i1}+2\varepsilon_{e}\right)}^{-1}+f_{2}\left(\varepsilon_{i2}-\varepsilon_{e}\right){\left(\varepsilon_{i2}+2\varepsilon_{e}\right)}^{-1}$$

where ε_i,1–2_ are the dielectric functions of the inclusion materials; *f*_1_ and *f*_2_ = (1 − *f*_1_) are the volume fractions of the materials under consideration. In this approach, the inclusions are optically ordered and disordered phases of these materials [29]. Note that for a fullerene–polymer blend, EMA is used multiple times. Namely, first, this approximation is used to mix P3HT and PCBM, in a 1:1 weight ratio. In turn, both P3HT and PC70BM are subjected to EMA to mix their ordered and disordered material phases. The proportion of the ordered phase is expected to increase as the annealing progresses. Therefore, the ellipsometric models of these materials need to be matched to the spectral dependences of the ellipsometric angles obtained before and after annealing at different temperatures.

For the layer of neat P3HT, the low- and high-ordered phases’ dielectric functions were fitted with a model containing five Leng–Lorentz oscillators, which are expressed by the following formula [40]:

(2) $$\varepsilon \left(E\right)=\varepsilon_{\infty \;}+\overset{N}{\underset{j=1}{\sum }}\left(\frac{C_{0i}}{E^{2}}\;\left[e^{i\beta_{j}}{\left(E_{gj}-E-i\Gamma_{j}\right)}^{\mu_{j}}+e^{-i\beta_{j}}{\left(E_{gj}+E+i\Gamma_{j}\right)}^{\mu_{j}}-2Re\left[e^{-i\beta_{j}}{\left(E_{gj}+i\Gamma_{j}\right)}^{\mu_{j}}\right]\;-2i\mu_{j}EIm\left[e^{-i\beta_{j}}{\left(E_{gj}+i\Gamma_{j}\right)}^{\mu_{j}-1}\right]\right]\right)+m_{0}E^{x_{0}}+ik_{0}.$$

In the case of a single critical point, *β* is the phase factor, *C*_0_ is the amplitude, *μ* is the order of the pole, *E_g_* is the critical point energy, $\Gamma_{j}$ is the broadening of the *j* oscillator, and *N* is the number of oscillators. In Equation (2), E=ℏω is the photon energy, where $\hbar =6.58211\cdot \;10^{-16}\;eV\cdot s$ is Dirac’s constant and ω is the frequency of light. Meanwhile, in the case of the layer of PC70BM, the disordered and ordered phases’ dielectric functions were described by four Tauc–Lorentz oscillators [45], with the following relationships [40]:

(3) $$\varepsilon_{2}\left(E\right)=\{\begin{array}{c} \frac{AE_{0}C{\left(E-E_{g}\right)}^{2}}{\left(E^{2}-E_{0}^{2}\right)+C^{2}E^{2}}\frac{1}{E};\;E>E_{g}\\ \;0\;;\;E\leq E_{g} \end{array}$$

(4) $$\varepsilon_{1}\left(E\right)=\varepsilon_{1}\left(\infty \right)+\frac{2}{\pi }P\overset{\infty }{\underset{E_{g}}{\int }}\frac{x\varepsilon_{2}\left(x\right)}{x^{2}-E^{2}}dx$$

where *ɛ*_1_ and *ɛ*_2_ are the real and imaginary parts of the dielectric function and *E_0_* is the peak transition energy, *C* is the broadening term, *E*_g_ is the energy gap, and *P* stands for the Cauchy principal part of the integral. An additional fitting parameter, $\varepsilon_{1}\left(\infty \right)$, has been included in Equation (4). The dielectric function components $\varepsilon_{1},\;\varepsilon_{2}$ and layer thickness *d* were determined using the Spectra Ray 3 software; note that *ε*_1_ + *iε*_2_ = (*n* + *ik*)^2^. Surface images taken in situ using an optical microscope during the thermal annealing of the tested samples are shown also in Figure 2.

**Figure 3:**
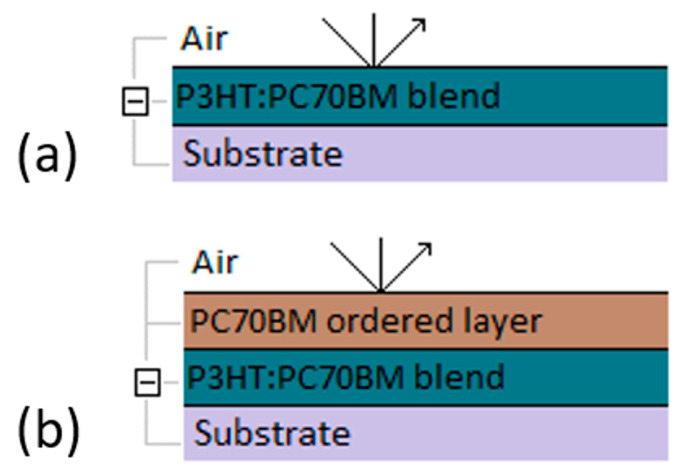
Ellipsometric models of P3HT–PC70BM blend films (**a**), before annealing and (**b**) after annealing with additional top layer of ordered phase of PC70BM.

After determining the dielectric functions of the considered materials, optical models of their layers can be defined, as shown in Figure 3a,b. It should be emphasized that the optical models of layers of pure materials and their mixtures in a 1:1 ratio are single-layer and homogeneous. The precipitation of PCBM aggregates on the surface of the blend during annealing was taken into account by adding a layer of ordered PCBM phase, the thickness of which is a parameter of the model.

**Figure 4:**
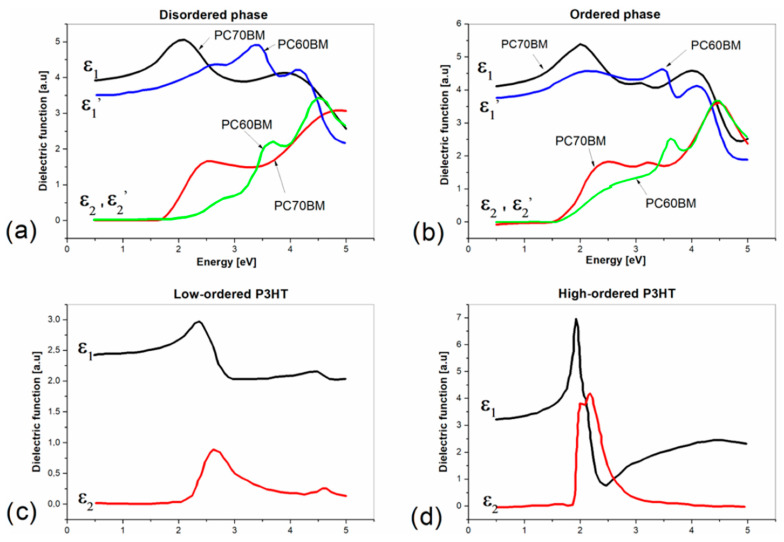
Dielectric function components (ε_1_—real part of dielectric function, ε_2_—imaginary part of dielectric function) of PC70BM, PC60BM (**a**,**b**) and P3HT (**c**,**d**).

The obtained components of the dielectric functions for P3HT and PC70BM are shown in Figure 4a–d.

**Figure 5:**
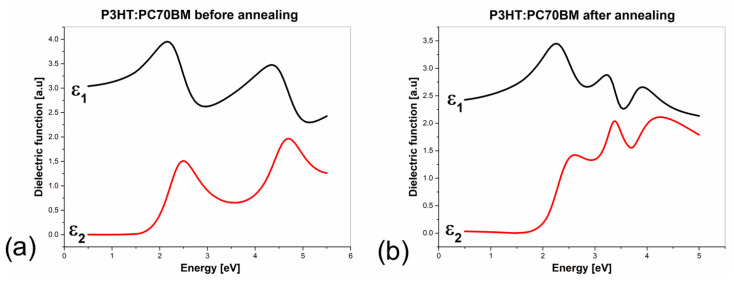
Dielectric function components of P3HT–PC70BM blend films before (**a**) and after annealing (**b**), where ε_1_ is the real part of dielectric function and ε_2_ is the imaginary part of dielectric function.

The components of the dielectric functions of P3HT–PC70BM blend films before and after annealing, determined using the presented model, are shown in Figure 5a,b, respectively, in energy scale. In this figure, the dielectric functions of PC60BM [9] are shown for comparison.

**Figure 6:**
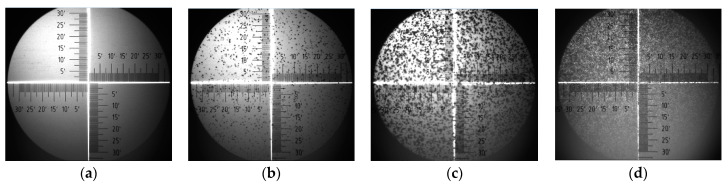
The surface of P3HT–PC70BM blend film, deposited onto substrate with 90 nm SiO_2_ layer, taken for 90 (**a**), 150 (**b**), 170 (**c**), and 210 °C (**d**).

Taken in situ during thermal annealing, optical images of the P3HT–PC70BM film showed that an ordered layer of PC70BM began to form on the surface regardless of the thickness of the silicon oxide layers that covered the substrates. Surface layers of ordered PC70BM began to form between 110 and 150 °C on blend films deposited onto silicon wafers, coated with 90, 300, and 400 nm SiO_2_, respectively. In Figure 6a–d are shown images of the blend surface deposited onto the Si substrate covered with a 90 nm thick SiO_2_ layer. The pictures were taken at 90, 150, 170, and 210 °C, respectively.

It is easy to notice that at 150 °C, the surface is already clearly covered with formed, numerous, single PC70BM aggregates. When the temperature is higher, the ordered phase of PC70BM begins to cover the surface more and more densely, and, finally, at 210 °C it covers the surface completely, and the crystallites are clearly larger in size.

Due to the surface changes, the ellipsometric model of the annealed polymer–fullerene blend films, including the lower- and higher-ordered phases of P3HT and PC70BM, was extended to account for the additional top layer of the ordered phase of PC70BM (Figure 3b). The fittings to the ellipsometric angles were taken for the lowest possible MSE, whose value was close to 1.

**Figure 7:**
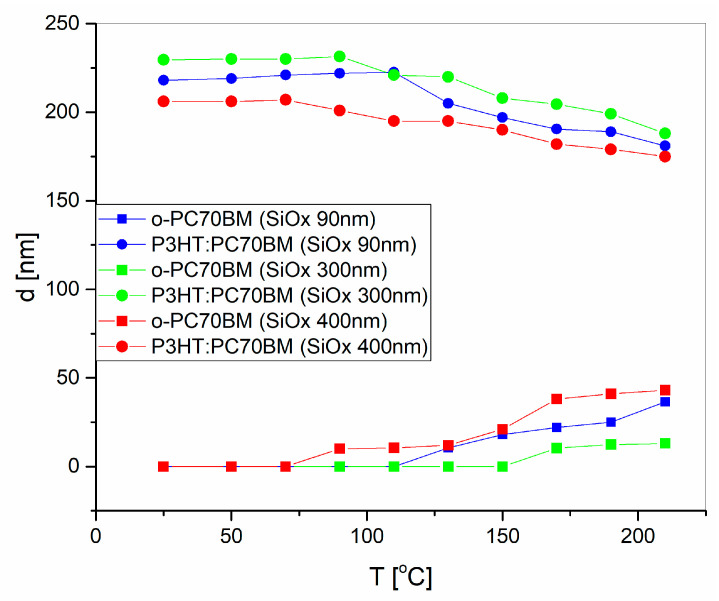
Changes in the thickness of the layer of the P3HT–PCBM mixture (circle symbols) and the layer of the ordered phase PC70BM (square symbols) after annealing at different temperatures.

The thickness as a function of temperature for films deposited onto silicon substrates, covered with 90 (blue symbols), 300 (green symbols), and 400 nm (red symbols) thick SiO_2_ layers, is presented in Figure 7. The blend films’ thickness, determined at 25, 50, 70, 90, 110, 130, 150, 170, 190, and 210 °C, is marked with circle symbols, whereas the corresponding layer thickness of the PC70BM ordered phase is marked with square symbols.

As can be seen from Figure 7, during thermal annealing, the thickness of the ordered PC70BM layer increases, while the thickness of the blend layer simultaneously decreases. This is because clusters of PC70BM form on the surface and grow using the material beneath them. It would be expected that in the entire temperature range in which the measurements were carried out, the thickness of the blend would start to change only when the formation of an ordered PC70BM agglomerate begins to be visible on the surface. The thickness of the ordered PC70BM layer, however, begins to change slightly earlier, which means that agglomerates begin to form earlier and are visible on the surface only as they grow. As the layer of ordered PC70BM grows deep into the deposited structure, the proportion of the PC70BM ordered phase, at the surface, increases as a result of two factors—the crystallization of the material inside the structure and the increasing thickness of the surface layer. Visible surface agglomeration, resulting from the crystallization of PC70BM under the influence of temperature, is related to changes in the morphology of the inner layer and the quality of the deposited material. The large diameter of the agglomerates particularly deteriorates the properties of the material in terms of its use as an active layer in optoelectronic devices.

## 4. Conclusions

The article presents the results of tests on thin films made of the P3HT–PC70BM blend. To study the optical, thermal, and structural properties of the films, two combined ellipsometric techniques were used, VASE and VTSE, as well as the in situ observation of changes in the appearance of the surfaces of the samples using an optical microscope. For the first time, the dielectric functions of the disordered and ordered phases of the PC70BM fullerene derivative were determined. For this purpose, the effective medium approximation was used, taking the dielectric functions of these phases as inclusions.

As a result of thermal annealing at a temperature of approximately 110 °C, the ordered PC70BM phase began to form on the surface of the P3HT–PC70BM blend layer. In order to account for this effect, the optical model used to describe the annealed polymer–fullerene film was extended with an additional layer of the ordered PC70BM phase. Treating the thickness of this layer as a model parameter significantly improved the fit to the experimental data. Moreover, it was found that the model described the changes that occurred on the surfaces of the layers with high accuracy and corresponded well to the images recorded in situ with an optical microscope.

The thickness of the silicon oxide layer on top of the substrates had no effect on the formation of the ordered PC70BM layer. In all tested cases, the thickness of the layer of the ordered PC70BM phase on the surface increased with increasing temperature, and the thickness of the layer of the P3HT–PC70BM mixture below it slightly decreased. It can be concluded that agglomerates of ordered PC70BM formed partially deep into the blend layer. The type of substrate also had no effect on the temperature at which the PC70BM layer began to precipitate.

## Data Availability

The data presented in this study are available on request from the corresponding author.
